# Recognition of Foal Nursing Behavior Based on an Improved RT-DETR Model

**DOI:** 10.3390/ani15030340

**Published:** 2025-01-24

**Authors:** Yanhong Liu, Fang Zhou, Wenxin Zheng, Tao Bai, Xinwen Chen, Leifeng Guo

**Affiliations:** 1 College of Computer and Information Engineering, Xinjiang Agricultural University, Urumqi 830052, China; lzyfhf@gmail.com (Y.L.); zwx2020@126.com (W.Z.); bt@xjau.edu.cn (T.B.); 2 Agricultural Information Institute, Chinese Academy of Agricultural Sciences, Beijing 100080, China; 3 Xinjiang Agricultural Informatization Engineering Technology Research Center, Urumqi 830052, China; 4 Ministry of Education Engineering Research Centre for Intelligent Agriculture, Urumqi 830052, China; 5 College of Information Science and Technology, Shihezi University, Shihezi 832000, China; zf_shzu@shzu.edu.cn; 6 Institute of Animal Husbandry Quality Standards, Xinjiang Academy of Animal Science, Urumqi 830011, China; 7 Xinjiang Intelligent Livestock Key Laboratory, Urumqi 830052, China

**Keywords:** feeding, artificial intelligence, foal suckling, behavior recognition

## Abstract

Foal nursing behavior is a crucial indicator of healthy growth. The mare being in a standing posture and the foal being in a suckling posture are important markers for foal suckling behavior. To enable the recognition of a mare’s standing posture and its foal’s suckling posture in stalls, this paper proposes an RT-DETR-Foalnursing model based on RT-DETR. The model employs SACGNet as the backbone to enhance the efficiency of image feature extraction. Furthermore, by incorporating a multiscale multihead attention module and a channel attention module into the Adaptive Instance Feature Integration (AIFI), the model strengthens feature utilization and integration capabilities, thereby improving recognition accuracy. Experimental results demonstrate that the improved RT-DETR achieves a best mAP@50 of 98.5%, increasing by 1.8% compared to the RT-DETR. Additionally, this study achieves real-time statistical analysis of the duration of the foal in the suckling posture, which is one of the important indicators for determining whether the foal is suckling. This has significant implications for the healthy growth of foals.

## Simple Summary

Timely monitoring and analysis of foal suckling behavior can provide valuable insights into foals’ physiological condition. A foal’s suckling posture and the mare’s standing posture during the nursing period are important prerequisites for the foal’s suckling behavior. Unlike manual observation and wearable devices, this study proposes a non-contact method using artificial intelligence (AI) vision technology to monitor the mare’s standing posture and the foal’s suckling posture. This method enables accurate recognition of both the mare’s standing posture and the foal’s suckling posture. Additionally, this study also implements real-time statistical analysis of the time the foal spends in the suckling posture. The proposed method offers a new perspective on equine reproduction for equestrian clubs and horse breeding enterprises, while also providing a supplementary approach for veterinarians and horse managers to detect early abnormalities in foal development.

## 1. Introduction

A foal’s nutrient intake from the mare’s milk is a critical factor known to influence the foal’s growth [1,2]. Under natural conditions, foals progressively stop suckling between 11 and 12 months of age [3]. The recognition and recording of foal suckling behavior and suckling time are of great significance for the healthy growth of foals. However, manual observation methods are difficult to sustain for long periods. Dealing with multiple foals, the manual approach faces significant challenges. Therefore, the use of advanced intelligent technologies to recognize foal suckling behavior is not only beneficial for the healthy growth of foals but also plays an important role in the automation level of foal breeding.

In recent years, researchers have achieved notable progress in intelligent recognition of animal behaviors. To recognize cattle behavior, several improved YOLOv8-based algorithms have been applied to recognize both basic and specific cattle behaviors [4,5,6,7]. These algorithms have contributed to the health and welfare of cattle while serving as one of the key approaches to enhancing farm management efficiency. Fuentes et al. [8] designed a framework based on artificial intelligence for monitoring the behavior of individual cattle through action recognition and tracking over time. This innovative approach enhanced the efficiency and effectiveness of farm operations. Cattle rumination behavior is strongly correlated with its health. Keypoint technology has been applied to recognize cattle rumination behavior [9], tracking physiological indicators during the rumination process, including chew count, rumination duration, and chewing frequency. This can help livestock managers monitor the health status of cattle in a timely manner. Bai et al. [10] proposed a X3DFast model for classifying dairy cow behaviors based on a two-pathway architecture. The technique obtains an accuracy of more than 97% for all individual dairy cow behaviors and a top-1 index of 98.49% for recognizing four dairy cow behaviors: walking, standing, mounting, and lying. Hao et al. [11] proposed a bottleneck attention-enhanced two-stream (BATS) method for behavior recognition in Jinnan cattle. This method can capture the spatial-channel dependencies in RGB and optical flow two branches, respectively, to extract richer and more robust features. The accuracy of behavior recognition reached 96.53%. Hua et al. [12] proposed an effective PoseC3D model for recognizing cattle locomotion behaviors, achieving a top-1 accuracy of 92.6%. In addition, wearable devices have been applied to cattle behavior recognition. Arablouei et al. [13] proposed a pioneering cross-device learning approach. By leveraging a pre-trained behavior recognition model from collar data to guide ear-tag model training, this method significantly reduces the need for manual labeling of ear-tag data. This breakthrough paves the way for personalized behavior recognition models using ear tags, requiring only brief periods of collar-based labeling per animal.

To recognize sheep behavior. Yu et al. [14] proposed a method for the recognition of ewe estrus based on a multi-target detection layer neural network. The accuracy of this method reached 98.56%. The results show that the method can meet the requirements of timely and accurate detection of ewe estrus behavior in large-scale mutton sheep breeding. Wan et al. [15] proposed a sheep face recognition model based on deep learning and bilinear feature fusion. The recognition accuracy of the algorithm is 99.43%, achieving individual recognition of sheep in complex environments while reducing the influence of pose and angle on recognition. Xu et al. [16] proposed a deep learning model that combines machine vision and time series analysis for recognizing aggressive behavior in group-housed sheep. The best accuracy was 93.38%. Gu et al. [17] proposed a two-stage (a detection stage and a classification stage) recognition method based on deep learning for sheep behavior. This method can recognize six behaviors (standing, feeding, lying, attacking, biting, and climbing). The mAP in the detection stage reached 98%, while the mAP in the classification stage was 94%. Pang et al. [18] proposed an Attention Residual Module (ARM) to aggregate the feature mapping between different layers of the CNN. This approach enables the general model of the CNN to be more adaptable to task-specific feature extraction. Using the ARM module, the recognition accuracy of the VGG16, GoogLeNet, and ResNet50 networks improved by 10.2%, 6.65%, and 4.38%, respectively. Zhang et al. [19] proposed a behavior identification method for housed sheep based on spatio-temporal information. ST-GCN was used to extract the spatial characteristics and time sequence features of behavior to accomplish the recognition of lying, ruminating, and other behaviors. The results show that after the improved keypoint selection strategy, the average accuracy of the sheep posture estimation model on the test set reached 95.6%, while the average accuracy of the behavior recognition model on the test set reached 99.9%.

To recognize the behavior of pigs, chickens, and other animals, Wang et al. [20] established an improved ResNAM network as a backbone network for pig face image feature extraction by combining an NAM (normalization-based attention module) attention mechanism and a ResNet model to probe non-contact open-set pig face recognition. The accuracy reached 95.28%. Zhuang et al. [21] developed a software system for monitoring pig feeding behavior, which can automatically recognize pigs and estimate their feeding and drinking times. Zhou et al. [22] proposed a method for individual pig identification using three-dimensional (3D) point. This approach is easy to integrate with functions such as body condition assessment and behavior recognition and is conducive to the development of precision livestock farming clouds of pigs’ back surface. Ji et al. [23] proposed a video behavior recognition method based on the Time Shift Module (TSM), which can automatically detect aggressive behavior occurring within a group of pigs. This method can recognize pig aggression effectively, which helps improve the use of automated management techniques in pig farming. Hao et al. [24] proposed a novel deep mutual learning-enhanced two-stream pig behavior recognition approach. The proposed model consists of two mutual learning networks, which include the red–green–blue color model (RGB) and flow streams. The accuracy reached 96.52%. In addition, the improved Cascade Region-Based Convolutional Neural Network was proposed for heat stress behavior recognition in yellow-feathered broilers [25]. A video mosaicing-based sensing method for chick behavior recognition on edge computing devices was proposed [26]. These studies have contributed to improving the welfare of chickens, particularly in terms of promoting healthy growth. Hu et al. [27] designed a Two-Stream ConvNet with Attention (TSCA) model; the recognition rate of the algorithm reached 92.04% and the positioning accuracy reached 78.4%. Nishioka et al. [28] proposed a Temporal Action Localization (TAL) method to recognize elephant behavior. The average precision of eating behavior detection using TAL was 85.3%.

Currently, compared to other livestock, there is not much research on horse behavior recognition. Martin-Cirera et al. [29] compared the performance of Transformers, LSTMs, and Bidirectional LSTMs for the classification of the behavioral time budget in horses based on video data. Experiments have confirmed that models with multiple inputs and outputs outperform models with a single input and a single output. However, this study did not systematically propose an advanced algorithm for the intelligent recognition of horse behavior. Liu et al. [30] proposed an improved SlowFast algorithm to recognize horse sleeping and feeding behaviors. This study also involved the recognition of various horse postures. The accuracy in identifying three postures—standing, sternal recumbency, and lateral recumbency—is 92.73%, 91.87%, and 92.58%, respectively. This algorithm also shows high accuracy in recognizing two behaviors—sleeping and eating—achieving accuracy of 93.56% and 98.77%. This study laid the foundation for the intelligent recognition of horse behaviors, but it did not delve into tracking the duration of horse behaviors. Based on research on intelligent recognition of behaviors in horses and other animals, this paper proposes an improved RT-DETR model for the intelligent recognition of foal suckling behavior, based on RT-DETR [31] and its improved algorithms [32,33,34,35,36]. The contributions of this paper are as follows:

(1) The SACGNet is proposed to enhance the backbone of RT-DETR, effectively improving RT-DETR’s ability to focus on important features in image data.

(2) The MCSA module is added to the AIFI of the RT-DETR model, strengthening the interaction between local and global features in the input and improving the performance of AIFI.

(3) A foal suckling behavior dataset is created, which can be used for recognizing foal suckling behavior in video data.

(4) Foal suckling behavior recognition and real-time suckling posture time statistics were implemented in video data, and the experimental results were analyzed.

## 2. Materials and Methods

### 2.1. Definition of Foal Nursing Behavior Labels

Foal nursing behavior refers to a foal obtaining nutrition by sucking the mare’s milk. After birth, the mare and the foal stay together [37]. In the experiment, it was observed that the foal can only suckle when the mare is in a standing posture. Therefore, this study focuses only on tracking the mare’s standing posture and the foal’s suckling posture when the foal is in the nursing state. As shown in Table 1, two labels are defined. When the object is the mare, we focus on its standing posture, and the label category is mare-standing. When the object is the foal, we focus on its suckling posture, and the label category is foal-suckling, indicating that the foal is in a suckling posture.

### 2.2. Experiment and Data Collection

The subjects of this study were mares and foals in the lactation period. The experiment was conducted at an experimental station (Xinjiang Ancient Ecological Park Akhal-Teke Horse Base in Urumqi, Xinjiang Uygur Autonomous Region, China). The data collection period was from May 2024 to June 2024. The video capture equipment used was smart camera devices, which were installed on the beams of the horse stalls. This non-contact data collection method does not disturb the daily life of horses. During the data collection period, continuous 24 h filming was conducted, with the video data having a resolution of 1912 × 1080 pixels and a frame rate of 30.0 frames per second. The video data we collected included various behavioral data from 8 foals (aged between 1 week and 8 months) and 8 mares from different time periods. The video durations ranged from 2 h to 8 h, with a total video volume of 2.24 TB.

### 2.3. Dataset Creation

**Figure 1:**
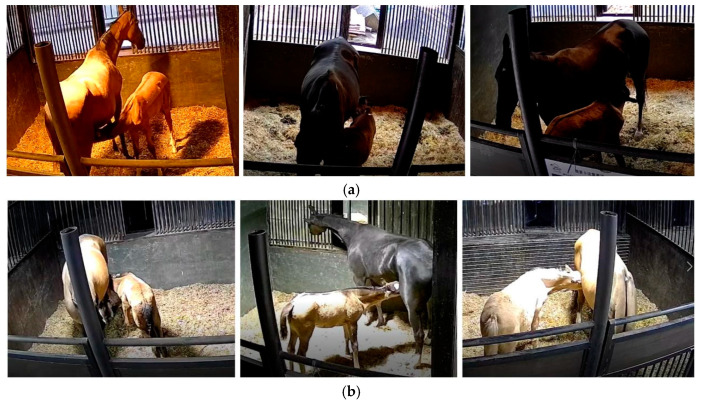
Dataset samples. (**a**) Sample examples of foal suckling behavior during the day. (**b**) Sample examples of foal suckling behavior at night.

After excluding video data with issues such as poor image quality, instances where the mare and the foal were not in the stall (e.g., when they were outdoors), and the foal did not exhibit suckling behavior for an extended period, we used video segmentation software to remove data with high redundancy (e.g., when the foal was suckling in the same position with a similar posture during certain time intervals) and obtained 32 video segments, with durations ranging from 10 s to 180 s. We employed keyframe extraction to obtain image data, resulting in a total of 3710 images. The ratio of the training, testing, and validation sets was 7:2:1. Figure 1 shows some samples from the dataset, which include foal suckling behavior under different lighting conditions, times, and angles. Additionally, our focus was solely on the mare’s posture and the foal’s behavior during suckling. Therefore, when labeling the data, we only labeled the foal’s suckling behavior and the mare’s posture. This approach allowed our research to stay focused on nursing behavior.

### 2.4. Model Implementation

#### 2.4.1. The Framework for Foal Nursing Behavior Recognition

**Figure 2:**
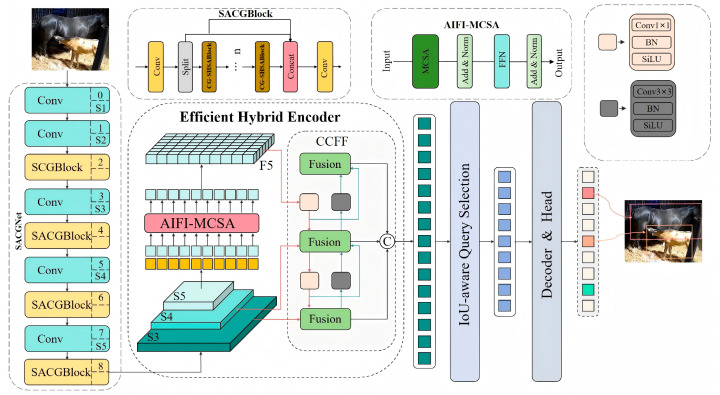
Overall architecture diagram of RT-DETR-Foal nursing.

The architecture of RT-DETR-Foalnursing is shown in Figure 2. The image data are processed by SACGNet for feature extraction, and the features from the last three stages of SACGNet (S3, S4, S5) are used as the input to the encoder. Then, within the Efficient Hybrid Encoder, the multiscale features are transformed into image feature sequences through the internal scale feature interaction (AIFI-MCSA) module based on multiscale multihead self-attention and the cross-scale feature fusion module (CCFM) based on convolutional neural networks. The IoU-aware query selection method is used to select a fixed number of image features as the initial object queries for the decoder. Finally, the decoder iteratively refines the object queries using auxiliary prediction heads to generate the bounding boxes and confidence scores.

#### 2.4.2. SACGNet

**Figure 3:**
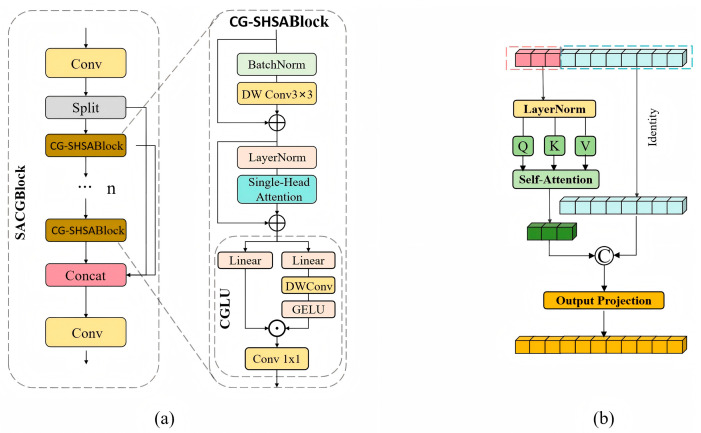
The structure of SACGBlock. (**a**) The overall structure of SACGBlock and the structure of the CG-SHSABlock module. (**b**) The structure of the single-head self-attention module.

SACGNet adopts a structure similar to the backbone of YOLOV8. At layer 0, the input image passes through an initial convolution to extract basic features and perform image downsampling. Then, it passes through 4 sets of Conv layers and the SACGBlock module. The SACGBlock module enhances feature representation through a single-head self-attention mechanism and a gating mechanism, which effectively improves the model’s ability to focus on important features. As the network depth increases, the spatial resolution gradually decreases while the number of channels increases, allowing the network to extract more complex features at deeper levels. The three stages {S3, S4, S5} of SACGNet were selected as the input for the efficient mixed encoder. Figure 3a shows the structure of the SACGBlock. First, Conv extracts the basic features from the input. Then, Split divides the feature map, with part of the features passing through multiple CG-SHSABlocks for feature extraction. Finally, the features processed by the CG-SHSABlock are concatenated with another portion of the input features via residual connections, thereby integrating local and global features, and the output features are produced through Conv.

The CG-SHSABlock is an efficient feature extraction module that incorporates the Convolutional Gated Linear Unit (CGLU) and Single-Head Self-Attention (SHSA). In the CGLU, part of the input features is passed through a depthwise separable convolution, followed by activation via Gaussian Error Linear Units (GELUs). Then, it is combined with another portion of the input features using a residual connection, ultimately forming a gated linear unit. This design allows each feature unit to acquire channel attention from the locally adjacent image features, thereby enhancing the local modeling capability and robustness of the model. Figure 3b shows the structure of SHSA. SHSA applies a single-head attention layer for spatial feature aggregation only to a subset of the input channels, while the remaining channels remain unchanged. The channel features processed by the single-head attention layer are concatenated with the unchanged input channel features via residual connection. Finally, the features are adjusted through Output Projection to generate the feature output. This design enables SHSA to effectively address the problem of head redundancy while improving training and inference efficiency.

#### 2.4.3. AIFI-MCSA

**Figure 4:**
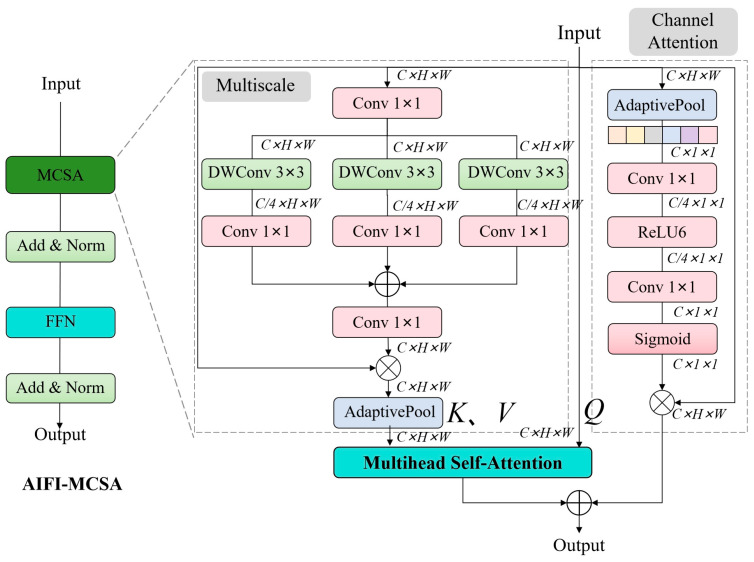
The structure of AIFI-MCSA.

As shown in Figure 4, the Multiscale Channel Self-Attention (MCSA) is incorporated into AIFI. MCSA integrates multiscale mechanisms, channel attention mechanisms, and multihead self-attention mechanisms. Its structure consists of two parts: one is the multiscale multihead self-attention module, and the other is the channel attention module. By combining multiscale convolution and self-attention mechanisms, it enhances the interaction of local and global features in the input, thereby improving the performance of AIFI.

The input feature X∈R^C×H×W^, where C represents the channel dimension, and H and W represent the image height and width, respectively. $X_{1}$ is the output after X is processed by MCSA. After channel processing, X is convolved using three parallel depthwise separable convolutions, followed by a 1 × 1 convolution. Then, summation operation, residual multiplication, and average pooling are performed to obtain $X_{m}$. Self-attention operation is then performed on $X_{m}$ and X to obtain $X_{1}$. The simplified operation process is as follows:

(1) $$X_{m}=AdaptivePool(X\times (Conv(\sum_{i=1}^{3}{DWConv}^{R_{i}}(X))))$$

(2) $$X_{1}=Attention(XW^{q},X_{m}W^{k},X_{m}W^{v})$$

$X_{2}$ is the output of X after passing through the channel attention module. In the channel attention module, X  undergoes a series of pooling, convolution, and activation operations to obtain $X_{n}$. Residual multiplication is then performed to obtain $X_{2}$. The simplified operation process is as follows:

(3) $$X_{n}=Sigmoid(Conv(ReLu6(Conv(Avgpool(X)))))$$

(4) $$X_{2}=X\times X_{n}$$

Finally, the features from these two branches are fused via a summation operation to obtain the output features of MCSA.

(5) $${X_{out}=X_{1}+X}_{2}$$

### 2.5. Foal Suckling Time Statistics

In the experiment, the timestamp variable $C_{s}$ is defined to record the frame count changes when the ‘foal-suckling’ label is detected. The initial value of $C_{s}$ is 0, and it increases by 1 when the ‘foal-suckling’ label is detected; otherwise, $C_{s}$ remains unchanged. The final value of $C_{s}$ represents the total number of frames where the ‘foal-suckling’ label is detected. Based on the video’s frame rate (FPS), the duration of the foal’s suckling posture can be calculated using the following formula:

(6) $$T_{sucking}=\frac{C_{s}}{FPS}$$

### 2.6. Evaluation Metrics

To evaluate the performance of the model, this study used precision (P), recall (R), average precision (AP), and mean average precision (mAP) as the evaluation metrics. During the evaluation process, the sample data were classified into true positives (TPs), false positives (FPs), true negatives (TNs), and false negatives (FNs) based on the true class and predicted class of each target object. n refers to the total number of categories.

(7) $$R=\frac{TP}{TP+FN}$$

(8) $$P=\frac{TP}{TP+FP}$$

(9) $$AP=\int_{0}^{1}P\left(R\right)dR$$

(10) $$mAP=\frac{\sum_{k=0}^{n}AP(k)}{n}$$

In Equations (7)–(10), recall (R) represents the proportion of correctly predicted targets among all targets, while precision (P) refers to the proportion of correctly predicted targets among all predicted targets. Average precision (AP) refers to the detection accuracy of each category, and mean average precision (mAP) refers to the average of the AP (average precision) values for all categories. These metrics were used to evaluate the overall performance of the model.

## 3. Experiments and Results

### 3.1. Experimental Environment

All model improvements and model training in this experiment were conducted in the same computer environment and configuration, as shown in Table 2.

### 3.2. Model Performance Analysis

#### 3.2.1. Performance Evaluation

**Figure 5:**
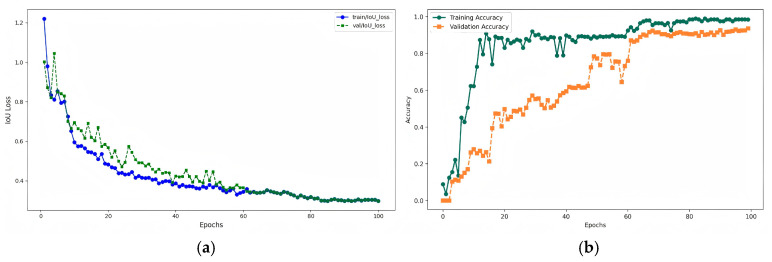
Model training performance graphs. (**a**) Loss function graph: the blue curve is the loss function of the training set, and the green curve is the loss function of the validation set. (**b**) Accuracy graph: the green curve is the accuracy of the training set, generated from the data during training. Accuracy is the mean precision calculated with an IoU threshold of 0.5. The orange curve represents the accuracy of the validation set, reflecting the model’s performance evaluation on the validation set using the weights obtained after training is completed.

The change curves of the IoU loss function on the training set and validation set during the training process of the improved model are shown in Figure 5a. Both loss functions generally show a downward trend, with a relatively slow decrease, indicating that the classification task is complex. However, after 60 epochs, the curves tend to stabilize, suggesting that the model, after learning for multiple epochs, can distinguish between different categories of targets.

In the experiment, we set the training epochs to 100, the initial learning rate to 0.0001, the optimizer to AdamW, and the weight decay to 0.0001. To more intuitively compare the performance differences of the model on the training set and validation set, we visualized the accuracy of the training set and validation set during the training process. As shown in Figure 5b, the accuracy of the training set converges quickly. When the number of epochs = 20, the training set accuracy increases rapidly and gradually stabilizes. However, the accuracy curve of the validation set is smoother and converges more slowly. This reflects that before the model has learned enough features, the accuracy on the validation set is not high. As the number of epochs increases, the model learns enough features, and the accuracy on the validation set improves. The highest accuracy of the training set is 98.5%, and the highest accuracy of the validation set is 93.6%.

#### 3.2.2. RT-DETR-Foalnursing vs. Other Models

Table 3 shows the accuracy of the proposed method in this study compared to other models. In handling the foal nursing behavior dataset, the model proposed in this study outperforms other models in terms of recognition accuracy. The mAP@0.5–0.95 (%) is higher than that of other models, reaching 77.7%. The mAP@0.5 (%) is 1.8% higher than RT-DETR. In addition, the weight size of RT-DETR-Foalnursing is the smallest.

#### 3.2.3. Ablation Experiment

Table 4 shows the results of the ablation experiments, √ indicates that the module is used, while × indicates that the module is not used. When SACGNet is the backbone, the model’s feature extraction ability is significantly improved, with mAP@50 increasing by 1.17%. Furthermore, after applying AIFI-MCSA, the model’s receptive field is enhanced, and mAP@50 increases by an additional 0.63% compared to the previous result. When AIFI-MCSA is applied alone, the model’s mAP@50 improves by 0.31%, validating the effectiveness of AIFI-MCSA. The experimental results indicate that the improvements carried out in this study effectively enhance the model’s detection accuracy for foal suckling behavior.

#### 3.2.4. Feature Learning Visualization

**Figure 6:**
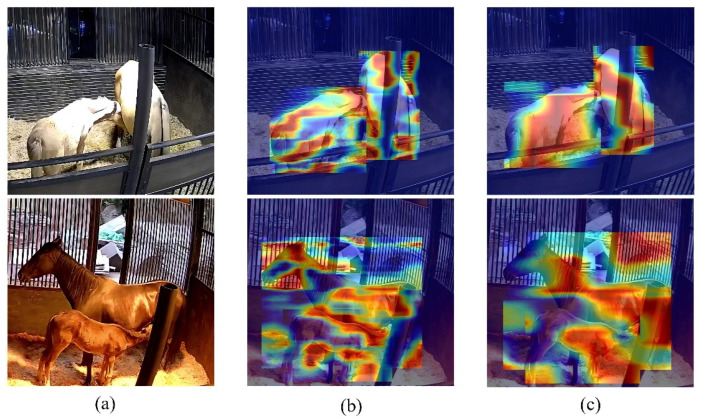
Samples of feature activation visualization heatmap for RT-DETR-Foalnursing. (**a**) The original image. (**b**) The feature activation output from the backbone. (**c**) The feature activation output from the Efficient Hybrid Encoder.

To observe the model’s performance more intuitively, we conducted heatmap visualization for RT-DETR-Foalnursing. The colors in the heatmap represent the density of feature points, with red indicating the highest feature density. As shown in Figure 6, after feature extraction through the backbone, the model has learned the features of the original image. After processing through the Efficient Hybrid Encoder, the model’s learning of features becomes more focused and denser. In Figure 6b, the heatmap is concentrated in the interaction area between the foal and the mare. This phenomenon is even more apparent in Figure 6c, indicating that the model can learn the behavioral features of foals and mares during the nursing period. In the heatmap at the bottom of Figure 6c, we can observe more clearly that a small portion of the heatmap is concentrated in areas outside the mare and the foal, which may lead to misclassification by the model. In the first row of images in Figure 6, the foal and the mare are in a well-lit environment, and the heatmap is almost entirely focused on the body parts of the foal and the mare. However, in the second row of images in Figure 6, the foal and the mare are in a dimly lit environment, with the overall background being backlit. While the heatmap is still concentrated on the foal and the mare, a small portion is focused on other areas. This indicates that, overall, the model is able to learn the behavioral features of foals and mares in different environments, but its performance slightly varies under different lighting conditions.

### 3.3. Recognition Result Analysis

#### 3.3.1. Foal Suckling Behavior Recognition Results

**Figure 7:**
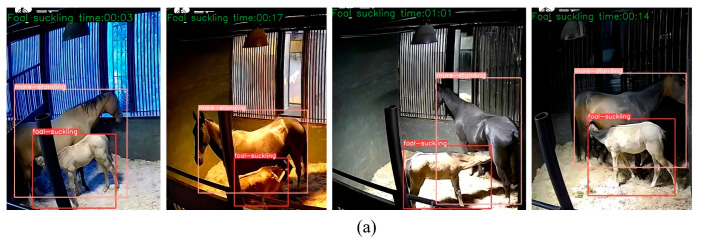

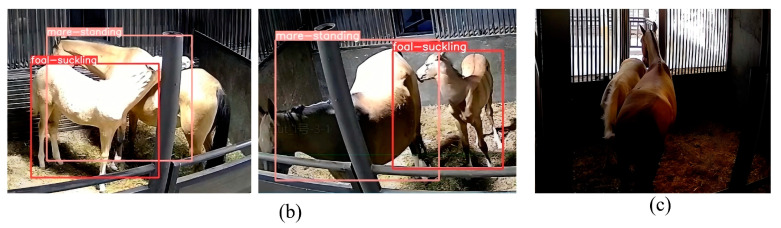
Samples of recognition results. (**a**) The correct situation, (**b**) misjudgment, and (**c**) missed detection, wherein a possible reason is that the foal is behind the mare.

Figure 7a shows a sample of foal nursing behavior recognition from the video data using the optimal weights trained with the RT-DETR-Foalnursing model. From the recognition results, the model proposed in this paper can detect foal suckling behavior in the stall under different angles and lighting conditions. Figure 7b shows a misjudgment, and the possible cause is that a certain movement of the foal in a standing posture is like suckling behavior. Figure 7c shows a missed detection, which may be due to insufficient feature learning of the foal suckling behavior by the model. Overall, misjudgment and missed detection are relatively rare.

In addition, we used the optimal model obtained from training to recognize five video clips, each lasting 60 s. The recognition results were then frame-extracted, with one frame extracted per second, resulting in five sets of frames, each containing 60 images, as shown in Table 5. We analyzed the recognition results, and the model demonstrated high accuracy, ranging from 95.0% to 98.3%, with low missed prediction rates (1.7% to 3.3%) and false prediction rates (0% to 3.3%). The second sample set achieved the highest accuracy (98.3%) with the fewest errors, while the third sample set had a higher false prediction rate and the lowest accuracy (95.0%). Overall, the model performed well with few missed and false predictions.

#### 3.3.2. Foal Suckling Behavior Time Statistical Analysis

**Figure 8:**
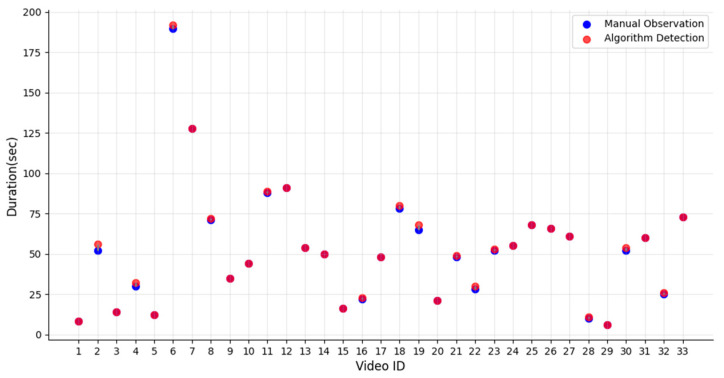
Comparison of foal suckling posture duration. The magenta points indicate that the blue and light red points completely overlap, showing that the values from automatic detection and manual observation are identical.

In the experiment, to verify the statistical method proposed in this paper for foal suckling posture time, 33 video segments were selected for manual inspection. These videos had already been processed by the method proposed in this paper, which recognized foal suckling behavior and automatically recorded the suckling time. As shown in Figure 8, most of the light red dots and blue dots overlap completely, with only a few light red and blue dots not perfectly overlapping (e.g., the dots for Video IDs 2, 4, 6, and 19), indicating that the automatically recognized duration matches the manually observed duration. Therefore, the method proposed in this paper enables intelligent recognition of foal suckling posture and real-time statistical analysis of suckling posture time.

#### 3.3.3. Foal Suckling Posture Time Statistics and Analysis

As shown in Table 6, the suckling posture times of foals with different IDs presented in the table are from different days, and not the same day. This data represents the suckling posture times of the foals in the stall and does not include outdoor suckling instances. We used the method proposed in this paper to conduct a preliminary analysis of the suckling posture time of eight foals. From the table, the suckling posture time varies among foals of different ages and breeds. The duration of each suckling posture event generally ranges from 5 s to 120 s, with very few instances exceeding 120 s. The suckling time is typically concentrated during the early morning hours (at those hours, the area around the stall is very quiet). The foals also suckle in the evening or close to midnight (foals are outdoors during the best sunlight of the day). The 2-week-old Akhal-Teke foals have the most frequent suckling posture, followed by the 1-month-old Thoroughbred foals. As foals age, the frequency of suckling posture gradually reduces. The primary reason is that as foals grow older, their caretakers provide supplementary food based on their physical development. Additionally, we observed that when older foals attempt to suckle, their mothers tend to move away or swat them with their tails to discourage suckling.

## 4. Discussion

### 4.1. Recognition of Foal Suckling Behavior in Backlit and Low-Light Environments

**Figure 9:**
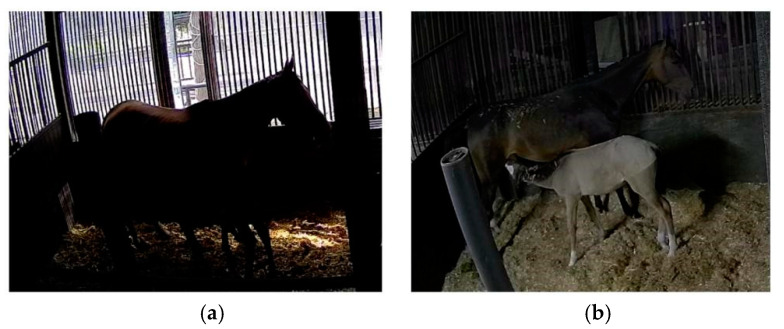
Samples of foal suckling behavior in backlit and low-light environments. (**a**) Backlit situation and (**b**) low-light situation.

As shown in Figure 9, in backlit conditions, strong backlighting causes the foal and the mare to appear very dark, or even completely blacked out, making the shape of the target unclear and increasing the difficulty of recognition. In low-light environments, the quality of the collected video data decreases and noise increases, which may affect the clarity of the target’s edges and the capture of fine details. The contours of the horses become blurred, especially under low lighting conditions, making it difficult for the detection algorithm to distinguish between the target and the background. Increasing the number of smart cameras at different angles or using thermal imaging cameras may be one of the solutions to address these issues.

### 4.2. Fine-Grained Recognition of Foal Suckling Behavior

**Figure 10:**
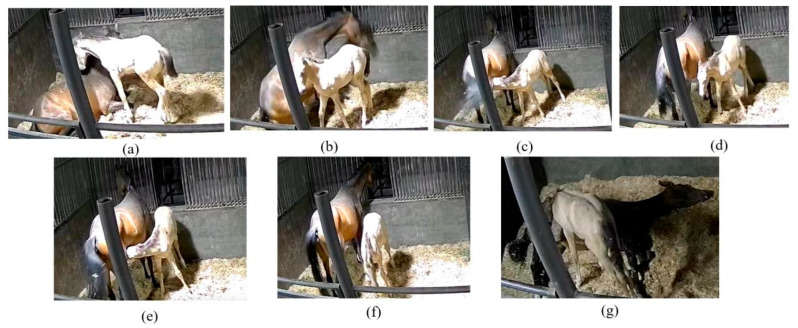
Examples of foal behavior during a feeding situation. (**a**) The foal approaches the mare. (**b**) The mare responds. (**c**) The foal begins suckling. (**d**) The foal pauses suckling. (**e**) The foal resumes suckling. (**f**) The foal finishes suckling. (**g**) The mare is in lateral recumbency and asleep, while the foal is suckling. This situation is extremely rare, but it has indeed occurred.

As shown in Figure 10, the definition of foal suckling behavior proposed in this study refers to the foal’s head moving toward the mare’s udder until the foal’s head moves away from the mare’s udder. This study does not focus on various stages such as the foal approaching the mare, the mare’s response, the foal suckling milk, and the foal pausing during suckling. By adopting a fine-grained recognition approach to distinguish between different suckling phases, actions, positions, postures, and so on, it is possible to more accurately capture and statistically analyze each detail of the foal’s suckling process, providing deeper insights and data support.

### 4.3. Statistical Analysis of Multiple Foals’ Suckling Time

This paper proposes a method for statistical analysis of foals’ suckling time, which currently enables real-time statistics of suckling time for a single foal in video data. However, if multiple foals are suckling simultaneously in the video data and their suckling end times differ, the proposed method will no longer be applicable. Additionally, the suckling time statistical method proposed in this paper relies on the FPS of the video data. If the FPS changes, it is necessary to redefine the FPS. By providing common FPS values in the algorithm, the issue of varying FPS values can be effectively addressed, enabling accurate statistical analysis of foal suckling time. However, if multiple foals suckle in the same video data, separately tracking the suckling times for each foal becomes a challenging task.

## 5. Conclusions

Applying advanced artificial intelligence algorithms to accurately identify a foal’s suckling posture in the stable is a critical condition for determining whether the foal is suckling. Accurate statistics and analysis of the duration and frequency of the foal’s suckling posture can bring benefits to the foal’s health. This study uses the DT-RTER-Foalnursing algorithm to intelligently recognize the standing posture of the mare and the suckling posture of the foal in video data during the nursing period, and implements real-time statistical analysis of the duration of the foal’s suckling posture. By accurately identifying and analyzing the foal’s suckling posture, more scientific data can be provided for equine reproduction, thereby improving breeding efficiency and success rates. It also assists veterinarians and horse managers in monitoring the health status of foals, while enabling equestrian clubs and breeding enterprises to identify potential health issues affecting foals and take timely action.

## Figures and Tables

**Table 1:** Definition of labels for foal nursing behavior.

Label	Object	Posture/Behavior	Description
Mare-standing	Mare	Posture	The four hooves of the mare touch the ground, supporting its body.
Foal-suckling	Foal	Posture	The foal stretches its head towards the mare’s udder, suckling milk.

**Table 2:** Experimental environment.

Configurations	Parameters
OS	ubuntu20.04
CPU	12 vCPU Intel(R) Xeon(R) Silver 4214R
GPU	RTX 3080 Ti(12 GB)
CUDA Version	Cuda 11.3
Memory	90 GB
Deep Learning Framework	PyTorch 1.10.0

**Table 3:** Model performance comparison.

Model	mAP@0.5 (%)	mAP@0.5–0.95 (%)	Weight Size (MB)
YOLOv5m	95.1	73.2	133
YOLOv8m	95.8	72.4	135
RT-DETR	96.7	75.1	113
RT-DETR-Foalnursing	98.5	77.7	107

**Table 4:** Results of ablation experiment.

Base Model	SACGNet		MCSA	Accuracy of Recognition		
	SHSA	CGLU		Mare-Standing	Foal-Suckling	mAP@50
RT-DETR	×	×	×	96.5	96.9	96.7
RT-DETR	√	×	×	98.85	95.69	97.27
RT-DETR	×	×	√	97.44	96.58	97.01
RT-DETR	√	√	×	98.91	96.83	97.87
RT-DETR	√		√	98.03	98.03	98.03
RT-DETR	√	√	√	98.98	98.02	98.5

**Table 5:** Sample prediction result analysis.

Group Number	Correct Predictions	Missed Predictions	False Predictions	Missed Rate	False Rate	Accuracy
1	58	1	1	1.7%	1.7%	96.7%
2	59	1	0	1.7%	0	98.3%
3	57	1	2	1.7%	3.3%	95%
4	58	2	0	3.3%	0	96.7%
5	58	1	1	1.7%	1.7%	96.7%

**Table 6:** Foal suckling time statistics.

ID	Age	Breed	Suckling Posture		
			Start Time (HH:MM:SS)	End Time (HH:MM:SS)	Duration (s)
1	2 weeks	Akhal-Teke	2:38:04	2:39:04	60
			2:46:19	2:46:34	15
			2:59:28	3:00:43	75
			4:29:34	4:29:40	6
			5:12:39	5:14:21	102
			6:09:10	6:10:06	56
			8:44:05	8:45:10	65
2	4 months	Akhal-Teke	6:50:36	6:51:06	30
			7:27:22	7:28:24	62
			19:49:03	19:50:25	82
			21:04:13	21:05:05	52
3	6 months	Akhal-Teke	0:16:14	0:17:22	68
			5:23:11	5:23:32	21
4	1 months	Thoroughbred	1:10:45	1:11:39	54
			4:00:54	4:02:33	99
			20:14:29	20:16:02	93
			21:53:18	21:54:58	100
			22:21:50	22:22:58	68
			23:58:28	23:59:38	70
5	2 months	Thoroughbred	2:40:39	2:42:47	128
			5:50:17	5:50:49	32
			8:13:42	8:13:56	14
			23:39:07	23:39:24	17
6	5 months	Thoroughbred	3:23:41	3:24:53	72
			3:45:02	3:45:57	55
			6:39:27	6:39:58	31
7	5 months	Thoroughbred	19:54:22	19:55:35	73
			20:03:02	20:03:55	53
			21:55:40	21:56:34	54
8	7 months	Thoroughbred	21:32:57	21:33:26	29
			22:57:19	22:58:29	70

## Data Availability

Data is available on request from the authors.
